# Dynamics of an Ongoing *Wolbachia* Spread in the European Cherry Fruit Fly, *Rhagoletis cerasi* (Diptera: Tephritidae)

**DOI:** 10.3390/insects10060172

**Published:** 2019-06-14

**Authors:** Martin Schebeck, Lukas Feldkirchner, Christian Stauffer, Hannes Schuler

**Affiliations:** 1 Department of Forest and Soil Sciences, University of Natural Resources and Life Sciences Vienna, BOKU, Peter-Jordan-Straße 82/I, A-1190 Vienna, Austria; martin.schebeck@boku.ac.at (M.S.); lukas.feldkirchner@gmail.com (L.F.); christian.stauffer@boku.ac.at (C.S.); 2 Faculty of Science and Technology, Free University of Bozen-Bolzano, Universitätsplatz 5, I-39100 Bozen-Bolzano, Italy

**Keywords:** endosymbiont, cytoplasmic incompatibility, bacterial spread, horizontal transfer, maternal transmission, reproductive manipulation, agricultural pest

## Abstract

Numerous terrestrial arthropods are infected with the alphaproteobacterium *Wolbachia*. This endosymbiont is usually transmitted vertically from infected females to their offspring and can alter the reproduction of hosts through various manipulations, like cytoplasmic incompatibility (CI), enhancing its spread in new host populations. Studies on the spatial and temporal dynamics of *Wolbachia* under natural conditions are scarce. Here, we analyzed *Wolbachia* infection frequencies in populations of the European cherry fruit fly, *Rhagoletis cerasi* (L.), in central Germany—an area of an ongoing spread of the CI-inducing strain *w*Cer2. In total, 295 individuals from 19 populations were PCR-screened for the presence of *w*Cer2 and their mitochondrial haplotype. Results were compared with historic data to understand the infection dynamics of the ongoing *w*Cer2 invasion. An overall *w*Cer2 infection frequency of about 30% was found, ranging from 0% to 100% per population. In contrast to an expected smooth transition from *w*Cer2-infected to completely *w*Cer2-uninfected populations, a relatively scattered infection pattern across geography was observed. Moreover, a strong *Wolbachia*-haplotype association was detected, with only a few rare misassociations. Our results show a complex dynamic of an ongoing *Wolbachia* spread in natural field populations of *R. cerasi*.

## 1. Introduction

Heritable bacterial endosymbionts are present in a broad range of arthropods with intriguing effects on the ecology and evolution of numerous species [1,2]. Endosymbionts are usually transmitted vertically from infected females to their offspring. By manipulating the reproduction through the induction of cytoplasmic incompatibility (CI), parthenogenesis, feminization, and male-killing, bacterial endosymbionts increase the fitness of infected females and enhance their frequency in host populations [3,4]. Certain endosymbionts are also able to provide fitness benefits protecting their host against RNA viruses or natural enemies [5,6]. Most endosymbionts are able to invade and adapt to new species by interspecific horizontal transmission [7,8,9,10].

One of the most common bacterial endosymbionts that can manipulate arthropod reproduction is *Wolbachia* [11]. This bacterium has been estimated to infect more than 50% of terrestrial arthropod species [1] and is present in numerous hexapods, crustaceans, chelicerates, and nematodes [11]. The most common reproductive manipulation is CI that is expressed when a *Wolbachia*-infected male mates with a *Wolbachia*-uninfected female, or with a female harboring a different *Wolbachia* strain, resulting in embryonic death of fertilized eggs [12].

A *Wolbachia* infection can influence the mitochondrial genetic structure of the host [13]. When a *Wolbachia* strain spreads into a population of uninfected individuals, the mitochondrial haplotype associated with the infected individual will hitchhike with the spreading endosymbiont [13,14,15,16,17]. As a result, an endosymbiont infection can affect the genetic population structure of arthropod species by reducing the diversity of mitochondrial haplotypes when infected individuals—associated with a specific mitochondrial haplotype—eliminate haplotypes associated with uninfected individuals [13].

The European cherry fruit fly, *Rhagoletis cerasi* (L., 1758) (Diptera: Tephritidae), is a widespread insect with a complex life history. In addition to a broad spectrum of evolutionary research questions [9,16,18,19,20], this tephritid is also of particular interest in applied fields [21]. It infests hosts of two different plant families, that is, *Prunus* spp. (Rosaceae) and *Lonicera* spp. (Caprifoliaceae) [18,20] and is therefore of significant human interest, as it is a severe pest of sweet and sour cherries [22,23].

*Rhagoletis cerasi* has a univoltine life cycle. In spring, prior to the ripening of fruits, adult flies emerge from their overwintering sites next to their natal host and usually do not disperse over long distances [21]. After mating on or close to host plants, usually one egg is deposited into a ripening fruit. These fruits are marked with specific pheromones to avoid an additional oviposition [24,25,26]. After embryonic development, larvae feed on fruits of their natal host plant. Last-instar larvae leave the fruits, dig into the soil, pupate, and overwinter in a diapausing state, completing their univoltine life cycle [21,27].

Moreover, *R. cerasi* has an intriguing reproductive biology. Crossing studies among European populations revealed strong incompatibility patterns from up to 98% when males from Southern and Central Europe mated with females from other European regions [18,19]. After the detection of *Rickettsia*-like organisms (RLOs) in reproductive organs [28], Riegler & Stauffer [29] described the presence of *Wolbachia* in this tephritid. While all individuals are infected by the same *Wolbachia* strain *w*Cer1, only populations from Southern and Central Europe are additionally infected by a second strain, *w*Cer2 [16,29]. The distribution of *Wolbachia* strains matches the incompatibility patterns reported by Boller et al. [19], which suggests that *w*Cer2 is causing this reproductive alteration [29].

**Figure 1:**
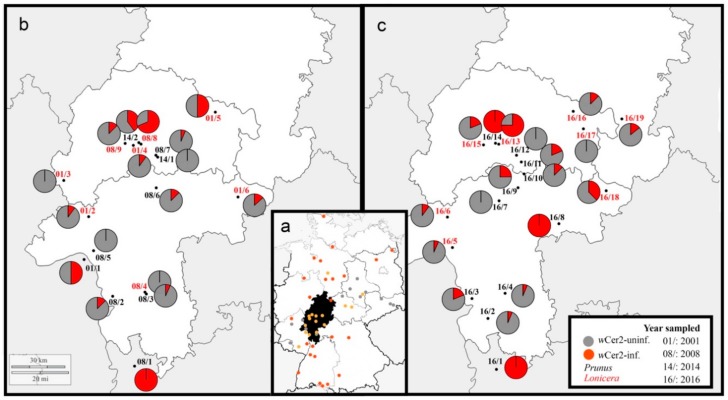
Geographic distribution of *Wolbachia* of *R. cerasi*: (**a**) Schematic overview of *Wolbachia* infections across Germany. Distribution of *w*Cer2-uninfected (grey dots), *w*Cer2-infected (red dots), and transitional populations with *w*Cer2-infected and *w*Cer2-uninfected flies (orange dots) sampled between 2000–2008, modified from [16]. Black-filled state represents Hesse, the central study site; (**b**) *w*Cer2 infection frequencies between 2001 and 2014 and (**c**) in 2016. Grey = proportion of *w*Cer2-uninfected flies, red = proportion of *w*Cer2- infected flies, black numbers represent flies collected from *Prunus* and red numbers represent flies collected from *Lonicera*.

Between southern and northern *R. cerasi* populations, there is a shift from completely *w*Cer2-infected populations to populations not infected with *w*Cer2 [29]. In these transitional zones, *w*Cer2-infected and *w*Cer2-uninfected flies coexist with gradients in infection frequencies (Figure 1a) [16,29,30]. Empirical studies combined with mathematic modelling showed that *w*Cer2 is currently invading *w*Cer2-uninfected *R. cerasi* populations [16,30]. A recent study of two *R. cerasi* transects in the Czech Republic and Hungary showed a smooth gradient from completely *w*Cer2-infected populations in the south to uninfected populations in the north and east, documenting a spatial spread of 1–2 km per generation [30].

Sequencing a part of the mitochondrial COI gene showed that *R. cerasi* exhibits a low genetic diversity in its European range, with only two haplotypes [16]. Comparison of haplotype of the fly and its *Wolbachia* infection status revealed a strong association between the endosymbiont and the mitochondrial haplotype—individuals of haplotype 1 (HT1) are *w*Cer2-uninfected, whereas *w*Cer2-infected flies are associated with haplotype 2 (HT2). Occasional misassociations have been reported from transitional zones as a result of intraspecific horizontal transfer and imperfect vertical transmission, highlighting the complex spread of this endosymbiont [16].

The invasion dynamics of *Wolbachia* in natural field populations are rarely studied [16,30,31,32,33]. To get detailed insights into the temporal and spatial spread of *Wolbachia* in *R. cerasi*, we performed a fine-scale sampling of 19 populations in Central Germany, where *w*Cer2 is currently spreading. By studying *Wolbachia* infection frequencies, assessing mitochondrial genotypes of the host, and comparing these results with historic data [16], we aim to get new insights into the dynamics of an ongoing *Wolbachia* spread under natural conditions.

## 2. Materials and Methods

### 2.1. Sampling and DNA Extraction

Samples were collected from infested fruits of *Prunus avium* and *Lonicera xylosteum* in July 2016. In total, specimens from 19 German locations were sampled: One site from the northern part of Baden-Württemberg, i.e., 16/1, and 18 populations from central-south Hesse, i.e., 16/2–16/19 (Table 1, Figure 1, Table S1). Individuals were collected from a single plant per location, either as larvae from infested fruits in the field or as pupae after emerging from cherries in the laboratory. To avoid the analysis of siblings, fruits from different parts of one tree/shrub were taken. Samples were stored in absolute ethanol at −20 °C. DNA was extracted from 16 individuals per location, except for the populations 16/5 (*n* = 15), 16/6 (*n* = 10), and 16/19 (*n* = 14; Table S1), using the GenElute Mammalian Genomic DNA miniprep kit (Sigma-Aldrich, St. Louis, MO, USA) following the manufacturer’s instructions.

### 2.2. Wolbachia Screening

Samples were PCR-screened for the presence of *w*Cer1 and *w*Cer2 using strain-specific primers targeting a part of the *Wolbachia* surface protein (*wsp*) [29,34,35]. PCR reactions were performed in a total volume of 10 µL, containing 1 mg/mL BSA, 2 mM Y-buffer (PeqLab/VWR, Erlangen, Germany), 800 µM dNTPs, 0.2 µM forward and reverse primer each, 0.5 U Taq polymerase (PeqLab/VWR), and 1 µL of template DNA. PCR conditions were 2 min at 94 °C, followed by 35 cycles of 94 °C for 30 s, 55 °C for 45 s, 72 °C for 1 min, followed by a final extension at 72 °C for 10 min.

PCR-amplified fragments were electrophoretically separated on a 2% agarose gel stained with GelRed Nucleic Acid Dye (Biotum, Hayward, CA, USA). As *w*Cer1 is fixed in European *R. cerasi* [16,29,30,35], screenings for this strain were performed to control for sufficient DNA quality. In order to avoid false-negatives, all results were confirmed by two independent PCR runs.

### 2.3. Mitochondrial Genotyping of R. cerasi

To study the association of *R. cerasi* mitochondrial haplotypes with the *Wolbachia* infection status, genotyping of the flies was performed by applying restriction fragment length polymorphism (RFLP) as used in [16]. In brief, a part of the mitochondrial COI gene was PCR-amplified using the primers Pat and Dick [36]. Following PCR (conditions were the same as described above), 10 µL of the PCR product was incubated with 2 U of *HaeIII* (Thermo Fisher Scientific, Waltham, MA, USA) at 37 °C for 3 h. Fragments were separated on a 2% agarose gel—HT2 is cut into a 342 bp and a 204 bp fragment, while HT1 remains undigested [16].

### 2.4. Comparison of Our Results with Historic Data

Eight out of the 19 *R. cerasi* populations studied here were already *Wolbachia*-screened and genotyped between 1999 and 2014 [16]: Dossenheim (16/1), Stockstadt (16/3), Ober-Ramstadt (16/4), Lich (16/12), Gießen (16/13, 16/14), Lahnau (16/15), and Alsfeld (16/16). To assess the spatial and temporal dynamics of *w*Cer2, infection frequencies of this strain of the various years were compared.

## 3. Results

### 3.1. Wolbachia Infection Frequencies

Screening of *R. cerasi* for the presence of *w*Cer2 revealed that 30.2% of samples (89 out of 295) were infected with this strain (Table 1, Figure 1c, Table S1). In three out of 19 locations, *w*Cer2 was fixed: In the southernmost site Dossenheim (16/1), and in two central locations Hailer (16/8) and Gießen/*Prunus* (16/14). In contrast, the three populations Rosbach (16/7), Lich (16/12), and Wallenrod (16/17) were completely *w*Cer2-uninfected. *w*Cer2 infection frequencies in the other 13 sites ranged from 6.3% in Bensheim (16/2) and Ober-Ramstadt (16/4) to 75.0% in Gießen/*Lonicera* (16/13) (Table 1, Figure 1c, Table S1).

*w*Cer2 frequencies of the *R. cerasi* populations studied here exhibit no distinct transitional infection pattern with smooth gradients from completely *w*Cer2-infected to entirely *w*Cer2-uninfected populations [30] (Figure 1c). The southernmost site Dossenheim (16/1) was completely *w*Cer2-infected, whereas the population in Bensheim (16/2), less than 30 km further north, showed a low infection frequency of 6.3%. *w*Cer2 frequencies in the adjacent locations Stockstadt (16/3) and Ober-Ramstadt (16/4), 14 km north-west and 17 km further north-east, respectively, ranged from 6.3% to 18.8% (Figure 1c). The *w*Cer2 infection frequency was similar in Erbenheim (16/5) and Idstein (16/6), 30 km and 50 km further north, with 6.7% and 10%, respectively. The population in Rosbach (16/7), however, was already completely *w*Cer2-uninfected. In contrast, 25% of the individuals from the population in Weckesheim (16/9), 13 km further north-west, were *w*Cer2-infected, whereas all individuals from Hailer (16/8), 30 km further south-east, were infected by *w*Cer2 (Figure 1c).

A similar pattern was found in the northern locations where a *w*Cer2-uninfected population (Lich, 16/12) was surrounded by two transitional populations 5 and 12 km apart, and just 14 km distant from the completely *w*Cer2-infected population in Gießen/*Prunus* (16/14) (Figure 1c).

### 3.2. Mitochondrial Genotyping of R. cerasi and Haplotype-Wolbachia Associations

**Figure 2:**
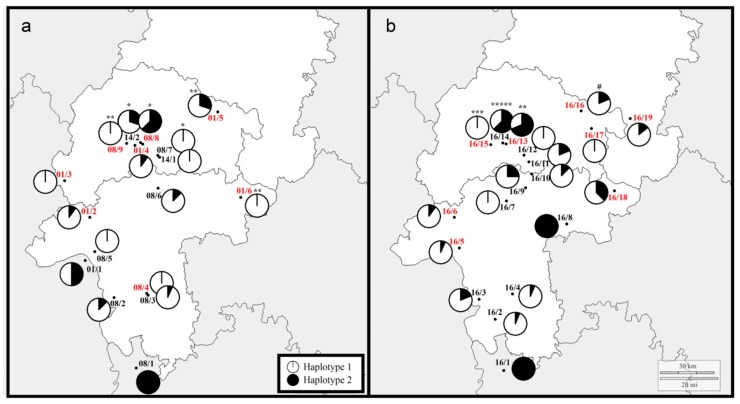
(**a**) Prevalence of haplotypes between 2001 and 2014 and (**b**) in 2016. White = proportion of individuals associated with HT1, black = proportion of individuals with HT2. Asterisks represent number of *w*Cer2-infected individuals associated with HT1, whereas the pound represents the only individual that was *w*Cer2-uninfected but associated with HT2.

As only two mitochondrial *R. cerasi* haplotypes across Europe have been described previously, we used an RFLP approach to determine the haplotype affiliation of each individual. Genotyping of flies showed that the *Wolbachia* infection status was strongly associated with the two mitochondrial haplotypes of the host (Figure 2b). Almost all *w*Cer2-uninfected individuals were associated with HT1 (205 out of 206). Just one individual from Alsfeld (16/16) was *w*Cer2-uninfected but associated with HT2. In contrast, 88.8% of *w*Cer2-infected *R. cerasi* were associated with HT2, whereas 10 *w*Cer2-infected individuals from three populations were associated with HT1 (Figure 2b).

Haplotype-*Wolbachia* misassociations were found in populations in geographically close proximity, i.e., Gießen/*Lonicera* (16/13), Gießen/*Prunus* (16/14), and Lahnau (16/15). In Gießen/*Lonicera* (16/13), two of the 12 *w*Cer2-infected samples were associated with HT1, whereas in Lahnau all three *w*Cer2-infected flies were associated with HT1. The most significant deviation was found in Gießen/*Prunus* (16/14), where all 16 individuals were *w*Cer2-infected, but only 11 (68.8%) were associated with HT2. 

### 3.3. Wolbachia Dynamics and Haplotype Associations in Time and Space

Out of the 19 *R. cerasi* populations studied here, eight had already been screened for their *Wolbachia* infection status and mitochondrial haplotypes between 1999 and 2014 [16]. We compared our results with these historic data to infer the dynamics of *Wolbachia* infection and the associated mitochondrial haplotype of the fly. *Rhagoletis cerasi* collected from Dossenheim (16/1)—where all individuals screened were infected by *w*Cer2—was already completely invaded by this strain in 1999 and 2008 (Table 2). In Stockstadt (16/3) and Lahnau (16/15), *w*Cer2 infection frequencies increased from 12.5% in 2008 to 18.8% in 2016 in both locations, however, *w*Cer2 frequencies did not differ significantly among these years (both locations: χ^2^ = 0.237, *p* = 0.434). In Ober-Ramstadt (16/4), the ratio of infected individuals remained constant—in 2008, one out of 15 flies (6.7%), and in 2016, one out of 16 flies (6.3%) was *w*Cer2-infected (χ^2^ = 0.002, *p* = 0.499). In 2008, the population in Lich (16/12) had a *w*Cer2 infection frequency of 6.3%, however, none of the flies were *w*Cer2-infected in 2014 (χ^2^ = 0.650, *p* = 0.339) and in 2016 (χ^2^ = 1.032, *p* = 0.297). A significant decrease of *w*Cer2 occurred in the *R. cerasi* population of Alsfeld (16/16), where 50% of individuals were *w*Cer2-infected in 2000, while in 2016, just 12.5% of the samples were infected by this strain (χ^2^ = 4.398, *p* = 0.036). Moreover, we found a potential effect of the host plant of the fly on its *Wolbachia* infection. In Gießen (16/13 and 16/14), flies collected from *Lonicera* had a significant increase of *w*Cer2 from 10% in 2001 to 68.8% in 2014 (χ^2^ = 8.547, *p* = 0.003). Comparing infection frequencies from the same site (16/13) in 2016 revealed a further increase in infection frequencies to 75% (χ^2^ = 0.155, *p* = 0.456). *Rhagoletis cerasi* collected from *Prunus* (16/14), however, was already completely *w*Cer2-infected.

Comparing *Wolbachia*-haplotype associations between different years revealed that *w*Cer2-infected flies associated with HT1 occurred in the same populations before (Figure 2a). Overall, the number of *w*Cer2-infected *R. cerasi* associated with HT1 increased in all three populations. In contrast, the population in Alsfeld (16/16), where one *w*Cer2-uninfected individual was associated with HT2, did not show this pattern in 2001. However, two out of five *w*Cer2-infected flies were associated with HT1 in 2001, a pattern that was not confirmed in 2016 (Table S2).

## 4. Discussion

Here, we studied the frequency of *w*Cer2 in *R. cerasi* in the central part of Germany, a region where this strain is currently spreading. Compared to previous work in this area [16], our fine-scale sampling and screening of 19 populations allowed us to characterize the spatial distribution of *w*Cer2 in this transition zone. Furthermore, comparing our results with historic data from the last two decades [16] allowed us to assess the temporal dynamics of this *Wolbachia* strain in certain populations. In contrast to our expectation of a transitional zone with smooth gradients from completely infected to entirely uninfected flies, as described in other parts of the species’ range [30], we found a surprisingly scattered pattern of *w*Cer2 infection frequencies across different populations. Comparison of the *Wolbachia* infection status and the mitochondrial haplotype of individuals showed a strong relationship, however, occasional misassociations suggest events of intraspecific horizontal transfer. Taken together, our data provide new insights into the ongoing *Wolbachia* spread in European *R. cerasi*.

### 4.1. Wolbachia Infection Frequencies in Time and Space

At least five *Wolbachia* strains have been described from *R. cerasi*, and one individual fly can harbor various strains [29,35]. The spread of the strain *w*Cer2 in Central Europe from south to north represents a rare event of an ongoing *Wolbachia* invasion in natural field populations [16,29,30]. By screening 295 individuals from 19 populations, we found an overall *w*Cer2 infection frequency of about 30%, ranging from 0% to 100% per population. Instead of gradual transitional zones between completely *w*Cer2-infected and entirely *w*Cer2-uninfected *R. cerasi* populations [30], a relatively scattered infection pattern was found. Populations completely infected with *w*Cer2 were in close proximity to locations with low *w*Cer2 infection frequencies. Our results, together with previous data [16,29,30], provide a comprehensive picture of an ongoing *Wolbachia* spread in natural field populations of *R. cerasi* and suggest that the spatial pattern of this endosymbiont infection is influenced by various factors, such as long distance migration of flies and/or passive movement with infested cherries.

Although *Wolbachia* is one of the best-studied bacterial endosymbionts, only a limited number of studies have given insight into its spatial dynamics under natural field conditions [16,30,31,37,38]. In addition to empirical observations, theoretical modelling can help to provide a basic understanding on the mode of *Wolbachia* spread [14,39,40]. One of the best studied examples of an ongoing *Wolbachia* spread in the field is the mosquito *Aedes aegypti,* artificially infected with the strain *w*Mel, that naturally occurs in *Drosophila melanogaster* [41,42]. *w*Mel was found to spread relatively slowly in release areas with a rate of 100–200 m per year [41]. Major factors influencing this spread are deceased fitness of transinfected mosquitoes and a low dispersal rate of the host [41]. This is in contrast to the estimated spread of *w*Cer2 in central Europe of 1–1.9 km per year [30], a spread that might benefit from the long adaptation of *w*Cer2 to its host—with expected low fitness costs—and the higher dispersal capacity of the fly. Both systems showed that human-mediated dispersal of insects can influence their long-range migration and can result in unexpected spatial patterns of host organisms and their associated symbionts [16,43].

Generally, *Wolbachia* that causes fitness costs in its host needs to reach a sufficiently high equilibrium frequency to get established in a host population [41]. Thus, infections in low frequencies do not result in an establishment of the bacterium [40]. In contrast, *Wolbachia* can be established in a host population even from very low initial infection frequencies by providing positive fitness effects to their host, as it was reported from *Drosophila simulans* in Australia [37] and California [31]. Although the spatial pattern of *w*Cer2 infection rates in Germany is different from other regions in the range of *R. cerasi* [16,29,30], our data suggest that a certain equilibrium infection frequency in a population is necessary for this strain to become established [16,30]. This was shown by the comparison of *Wolbachia* infection frequencies at the same sites among different years. For example, in the locations Ober-Ramstadt (16/3), Stockstadt (16/4), Idstein (16/6), Lich (16/12), and Lahnau (16/15), infection frequencies remained at low levels over a period of eight to 15 years. In contrast, in Gießen/*Lonicera* (16/13), *w*Cer2 infection frequencies of *R. cerasi* collected from honeysuckle increased rapidly from 10% in 2001, to 68.8% in 2008 [16], and to 75% in 2016. We assume that *w*Cer2 reached a sufficiently high infection frequency over time, and we expect that this strain will get fixed in the following years. The importance of an equilibrium infection frequency is further supported by the absence of intermediate infection frequencies over the sampling years, as it was found in almost all locations.

In Gießen/*Prunus* (16/14), however, *R. cerasi* was already completely *w*Cer2 infected. This suggests an influence of the fly’s host plant on the *Wolbachia* infection dynamics. Some lines of evidence propose host plant-related differentiation patterns in *R. cerasi*. For example, individuals from *Prunus* and *Lonicera* show slightly different eclosion times in spring, maybe a response to a differing fruiting phenology of the host [44]. Potential ecological differentiation between host ecotypes of *R. cerasi* infesting cherry or honeysuckle [18,20] might reduce gene flow between different populations, influencing the *Wolbachia* infection dynamics. A contrasting pattern was observed in the northernmost location Alsfeld (16/16) where the ratio of *w*Cer2 infections decreased significantly from 50% in 2000 to 12.5% in 2016. Since none of the *w*Cer2-uninfected individuals had the mitochondrial haplotype HT2, we exclude events of occasional loss of *w*Cer2 as a reason for the decrease of this strain over time. The accidental anthropogenic introduction of *w*Cer2-uninfected flies into this region, for example, via trade with *R. cerasi*-infested cherries, could explain this finding.

### 4.2. Wolbachia Infection and Mitochondrial Haplotype of the Host

The genetic structure of European *R. cerasi* on the mitochondrial level is generally low, reflected by the presence of only two haplotypes [16]. This low genetic diversity might be the result of a previous *Wolbachia* sweep by the strain *w*Cer1. Schuler et al. [16] hypothesized that a few individuals of *R. cerasi* with HT1 got previously infected by *w*Cer1 via horizontal transmission. This strain might have provided fitness benefits to the fly and swept through host populations, replacing all other haplotypes. Later, HT2 evolved, acquired *w*Cer2 horizontally, and is now hitchhiking through European populations [16].

The mitochondrial haplotypes are tightly associated with *Wolbachia*, where *w*Cer2-uninfected flies are associated with HT1 and *w*Cer2-infected individuals with HT2. In our study, out of 295 samples, only 11 had a different *Wolbachia*-haplotype association. In 10 cases, these misassociations represented a *w*Cer2-infection in individuals with HT1. This finding can be a result of intraspecific horizontal transfer [45]. For example, parasitoid wasps are known to be a potential source of horizontal transmission among species [16,45,46,47,48]. Parasitoid species of *R. cerasi* were found to be infected with *Wolbachia*. Their role in horizontal transmission between and within fly species, however, has yet to be studied [45]. An additional mechanism of horizontal transfer might be via cannibalism among *R. cerasi* larvae [45]. Although multiple larvae within one cherry are uncommon [26], under certain environmental conditions [26] or when resources for oviposition are scarce, several larvae might develop in one fruit. In this case, a larva could acquire *Wolbachia* by feeding on a co-occurring larva but would remain associated with its mitochondrial haplotype.

Since all cases of a *w*Cer2-infection in HT1 flies were found exclusively in transitional populations (where flies with both *Wolbachia* infection types are present), this phenomenon was interpreted as transient, where HT1 is assumed to be lost in populations completely invaded by *w*Cer2 [16]. We found just three populations in a restricted area—in Gießen (16/13 and 16/14) and Lahnau (16/15)—with individuals belonging to HT1 harboring *w*Cer2. The comparison with historic data from 2001, 2008, and 2014 [16], however, showed a general increase of individuals with these misassociations. In the location Gießen/*Prunus* (16/14) where *R. cerasi* is completely *w*Cer2-infected, 31% of the individuals were associated with HT1. Subsequent studies are needed to understand if these flies can form a stable co-existence of *w*Cer2 and HT1 or if they will be lost in future generations, as simulated by Schuler et al. [16].

Finally, one single *w*Cer2-uninfected individual associated with HT2 was found. This suggests a potential case of unsuccessful maternal transmission of *Wolbachia*. The low occurrence of these misassociations reflects a strong CI-inducing effect of *w*Cer2 with nearly perfect transmission from females to their offspring [16,19].

## 5. Conclusions

Our screening of the European cherry fruit fly with focus on the CI-inducing strain *w*Cer2 provides new insights into this unique endosymbiont-host system. In contrast to an expected smooth transition and continuous gradient of infection frequencies from *w*Cer2-infected to completely uninfected *R. cerasi* populations [30], we found a rather scattered geographic infection pattern of *w*Cer2 infections. Our combined analysis of the *Wolbachia* infection status and the associated host genotype show a highly complex picture of just partially increasing *w*Cer2 frequencies, possibly shaped by endosymbiont losses, intraspecific horizontal transmission events, and potential anthropogenic effects.

## Figures and Tables

**Table 1:** Infection frequencies of *w*Cer2 across different *R. cerasi* populations and ratios of *w*Cer2-infected flies associated with haplotype 2 (HT2) per population (* = populations with *Wolbachia*-haplotype misassociations).

Population #	Location	% *w*Cer2	% *w*Cer2/HT2
16/1	Dossenheim	100	100
16/2	Bensheim	6.3	6.3
16/3	Stockstadt	18.8	18.8
16/4	Ober-Ramstadt	6.3	6.3
16/5	Erbenheim	6.7	6.7
16/6	Idstein	10.0	10.0
16/7	Rosbach	0.0	0.0
16/8	Hailer	100	100
16/9	Weckesheim	25.0	25.0
16/10	Utphe	12.5	12.5
16/11	Langsdorf	18.8	18.8
16/12	Lich	0.0	0.0
16/13	Gießen/*Lonicera*	75.0	62.5 *
16/14	Gießen/*Prunus*	100	68.8 *
16/15	Lahnau	18.8	0.0 *
16/16	Alsfeld	12.5	12.5
16/17	Wallenrod	0.0	0.0
16/18	Schlüchtern	37.5	37.5
16/19	Grossenmoor	14.3	14.3

**Table 2:** Comparison of *w*Cer2 infection frequencies among the years 1999 (99), 2001 (01), 2008 (08), and 2016 (16).

Location	Population #	Host	Year	*n*	% *w*Cer2
Dossenheim	99/1	*Lonicera*	1999	10	100
	08/1	*Prunus*	2008	16	100
	16/1	*Prunus*	2016	16	100
Ober-Ramstadt	08/3	*Lonicera*	2008	15	0
	08/4	*Prunus*	2008	15	6.7
	16/4	*Prunus*	2016	16	6.3
Stockstadt	08/2	*Prunus*	2008	16	12.5
	16/3	*Prunus*	2016	16	18.8
Lich	08/7	*Prunus*	2008	16	6.3
	14/1	*Prunus*	2014	10	0
	16/12	*Prunus*	2016	16	0
Gießen	01/4	*Lonicera*	2001	10	10
	08/8	*Lonicera*	2008	16	68.8
	16/13	*Lonicera*	2016	16	75
	16/14	*Prunus*	2016	16	100
Lahnau	08/9	*Lonicera*	2008	16	12.5
	16/15	*Prunus*	2016	16	18.8
Alsfeld	01/5	*Lonicera*	2001	10	50
	16/16	*Lonicera*	2016	16	12.5

## Supplementary Materials

The following are available online at https://www.mdpi.com/2075-4450/10/6/172/s1, Table S1: Detailed overview on the *Wolbachia* infection status and mitochondrial haplotypes of *R. cerasi*, Table S2: Overview on historic data on the *Wolbachia* infection status and the mitochondrial genotypes of *R. cerasi*.
